# mRNA export: threading the needle

**DOI:** 10.3389/fpls.2013.00059

**Published:** 2013-03-22

**Authors:** Ouassila Gaouar, Hugo Germain

**Affiliations:** Groupe de Recherche en Biologie Végétale, Département de Chimie et Physique, Université du Québec à Trois-RivièresTrois-Rivières, QC, Canada

**Keywords:** mRNA export, ALY, MOS11, CIP29, TAF15b, TAFs, RNA helicase, snc1

## Abstract

After mRNA biogenesis, several proteins interact with the messenger to ensure its proper export to the cytoplasm. Some of these proteins will bind RNA early on, at the onset of transcription by RNA polymerase II holoenzyme, while others will join later for downstream processing steps, such as poly-adenylation or splicing, or may direct mRNA ribonucleoprotein particle migration to the nucleopore. We recently discovered that *Arabidopsis* plant knockout for the protein MOS11 (MODIFIER OF SNC1, 11) partially suppresses autoimmune responses observed in the TNL-type [TIR/NBS/LRR (Toll-interleukin-like receptor/nucleotide-binding site/C-terminal leucine-rich repeat)] R gene gain-of-function variant *snc1* (*s*uppressor of npr1-1, constitutive 1). This suppression of resistance to pathogens appears to be caused by a decrease in nuclear mRNA export in *mos11-1 snc1* plants. In humans, the putative ortholog of MOS11, CIP29 (29-kDa cytokine-induced protein), interacts with three proteins that are also involved in mRNA export: DDX39 (DEAD-box RNA helicase), TAF15 of the FUS family (FUSED IN SARCOMA), and ALY (ALWAYS EARLY), a protein implicated in mRNA export in mammalian systems. These proteins have received very little attention in plants. Here, we will discuss their particularities and role in mRNA export and biotic stress.

## INTRODUCTION

Messenger RNA export is a tightly regulated process, unique to eukaryotes, that enhances control over the timing and level of translation. Albeit mRNA export is unidirectional, unlike nucleocytoplasmic protein trafficking, it represents a more complex process. Before mRNA can actually be led through the conduit formed by the nucleopore (NP) across the nuclear envelope (NE), it must be adequately processed and guided toward the NP basket. As mRNA elongates, it is loaded with proteins; some of them will mark intron–exon junctions, helicases will keep mRNA unwound, the spliceosome complex will bind to single-stranded nucleic acid, some will induce mRNA cleavage in its 3′UTR so that it can then be 3′-poly-adenylated and bound with more proteins that recognize the poly-A tail. Additional proteins will serve as guides to direct mRNA to the NP. This association of proteins and one mRNA molecule is known as the messenger ribonucleoprotein particle (mRNP). In fact, mRNP thickness has been evaluated to be approximately 5–7 nm (Batisse et al., 2009). In comparison, the free, double-stranded DNA molecule has a width of 2 nm, which increases to 10 nm when DNA is bound to nucleosomes. Hence, the traditional representation of a free and linear mRNA molecule generally illustrated in textbooks does not depict reality; rather mRNP is virtually the size of the double-stranded DNA molecule bound to nucleosomes.

Messenger ribonucleoprotein particle does not journey from the nucleoplasm to the cytoplasm by linear progression. As Siebrasse et al. (2012) beautifully demonstrated recently by light sheet microscopy, bottleneck of mRNP export occurs on the nuclear side of the NE, more precisely at the NP basket. Accumulation of mRNP at this precise location is indicative of intensive mRNP remodeling required to remove mRNP nuclear proteins that will not engage in NP conduit along with mRNA during its migration toward the cytoplasm. The bulk of mRNP is exported by proteins of THO/TREX (transcription-export) complex (Strasser et al., 2002). However, the unidirectionality of transport is conferred, on the cytoplasmic side, by a RNA helicase that unloads the mRNP of key transport-related proteins, preventing its return to the nucleus (Tran et al., 2007; Stewart, 2010). TREX complex is highly conserved across species and orchestrates several steps between mRNA synthesis and export (Katahira, 2012).

Knowledge of mRNA export has been garnered in mammalian species, *Drosophila* and yeast. However, this crucial aspect of cellular biology and translation regulation has received very little attention in plants. Genetic screens designed to modify the autoimmune phenotype in the TNL-type [TIR–NBS–LRR (Toll-interleukin-like receptor/nucleotide-binding site/C-terminal leucine-rich repeat)] R gene gain-of-function variant *snc1* (*suppressor of npr1-1, constitutive 1*) have identified a number of proteins involved in nucleocytoplasmic transport (reviewed in Monaghan et al., 2010). MOS2, discovered in a *snc1* modifier screen, is the first protein potentially involved in mRNA export (Zhang et al., 2005). It encodes a nuclear protein of unknown function that possesses a RNA-binding domain. The G-patch found in MOS2 is also observed in eukaryotic RNA-processing proteins. The single *mos2* mutant displays enhanced disease susceptibility to *Pseudomonas syringae maculicola* (*P.s.m.*) and Avr-containing *Pseudomonas* strains, indicating that MOS2 is involved in both basal and effector-triggered immunity (Zhang et al., 2005). Interestingly, MOS2 was recently found to be sumoylated (Miller et al., 2010). Sumoylation appears to be a functional regulatory key of proteins involved in mRNA export and processing (Meier, 2012). A number of studies targeting SUMO-enriched fraction or yeast two-hybrid with SUMO E2 or SUMO protease EARLY IN SHORT DAYS4 (ESD4) as baits discerned a significant number of proteins involved in RNA splicing and processing, DEAD/DEAH-box RNA helicases, and proteins with RNA-recognition motifs (Budhiraja et al., 2009; Elrouby and Coupland, 2010; Miller et al., 2010). MOS3 (also called SAR3/AtNup96), another protein discovered in *snc1* modifier screen (Zhang and Li, 2005), is an integral NP component of the conserved Nup107–Nup160 complex shown to be required for mRNA export (Parry et al., 2006). Similarly to *mos2*, single *mos3* mutant plants display enhanced disease susceptibility to *P.s.m.* and Avr-containing *Pseudomonas* strains linking MOS3 to both basal and effector-triggered immunity (Zhang and Li, 2005).

We recently reported the discovery of a novel *snc1* modifier, naming it *m*odifier of snc1, 11 (*mos11*), a suppressor of mRNA export (Germain et al., 2010). Unlike most previously identified *mos* mutants, including *mos2* and *mos3*, single *mos11* mutant does not display enhanced disease susceptibility, indicating that its role in immunity, observed in suppression of the *snc1* autoimmune phenotype, may be limited to R gene-mediated resistance. Although cellular function of the putative homolog of MOS11 in mammalian systems (CIP29, 29-kDa cytokine-induced protein) was not established, the physical interactors encountered in human cells are consistent with a role in mRNA export. In this review, we focus on MOS11 and interactors of the homolog of MOS11, found in non-plant systems. Since none of them has been described in plants, we will hypothesize their putative involvement in mRNA export and innate plant immunity.

## MOS11

*mos11* mutant was initially identified by T-DNA tagging in the *snc1* background. Typically, *snc1* plants are dwarfs that have elevated salicylic acid levels, constitutive *pathogenesis-related* genes expression and enhanced resistance to *Hyaloperonospora arabidopsidis* Noco2 and virulent *Pseudomonas syringae* strains. *mos11 snc1* plants do not fully revert to wild type-looking plants; *mos11* only partially suppresses the molecular and morphological features of *snc1* mutants. With inverse polymerase chain reaction, we identified a T-DNA insertion in At5g02770 and confirmed, by complementation with the wild type At5g02770 sequence, that this T-DNA insertion was causing the *mos11 snc1* phenotype (Germain et al., 2010). At5g02770 encodes a small protein of unknown function, and its sequence is unique in *Arabidopsis*. The human protein CIP29 (**Table 1**) is the closest homolog with a putative function found with BlastP (Fukuda et al., 2002). Insight into its putative role came with the identification of two yeast two-hybrid interactors of CIP29: the DEAD-box RNA helicases BAT1 and DDX39 (DEAD-box RNA helicase; Leaw et al., 2004). Patches of positively charged residues in CIP29 (and MOS11) indicate affinity for DNA or RNA and *in vitro* assays have demonstrated that CIP29 binds RNA (Sugiura et al., 2007). The observation that the RNA helicase activity of DDX39 was greatly enhanced in the presence of CIP29 provided a molecular role for CIP29 (Sugiura et al., 2007). In addition, these authors demonstrated that DDX39 could immunoprecipitate both CIP29 and ALY (ALWAYS EARLY). Dufu et al. (2010) established that recruitment of CIP29 to mRNA is capping- and splicing-dependent, positioning the role of CIP29 as a post-splicing event. Finally, the yeast ortholog of CIP29, Tho1 (Transcriptional defect of Hpr1 by overexpression), can suppress the RNA export defect of *hpr1δ* when overexpressed (Jimeno et al., 2006). Using whole mount *in situ* total mRNA localization, we clearly determined that *mos11* and *mos11 snc1* plants manifest decreased mRNA export and accumulate poly-A mRNA in their nuclei (Germain et al., 2010). Surprisingly, despite a rather dramatic decrease in mRNA export, plant morphology appears to be virtually identical to that of wild type plants, indicating that all mRNA probably eventually reach the cytosol. The important transcriptional reprograming responsible for *snc1* elevated defense is likely to be dimmed in this mutant that shows nuclear mRNAs accumulation.

**Table 1:** CIP29 interacting proteins and their putative orthologs in *Arabidopsis*.

Human gene (NCBI gene ID)	*Arabidopsis* ortholog (TAIR gene ID)	Putative function	Reference
*CIP29* (84324)	*MOS11* (At5g02770)	Activation of DDX39	Sugiura et al. (2007), Germain et al. (2010)
*TAF15* (8148)	*AtTAF15b* (At5g58470)	Unknown	Sugiura et al. (2007)
*DDX39B/UAP56/BAT1* (7919)	*AtRH15* (At5g11170) *AtRH15b *(At5g11200)	DEAD-box RNA helicase	Aubourg et al. (1999)
*ALY/REF/BEF/THOC4* (10189)	*AtALY1* (At5g59950) *AtALY2* (At5g02530) *AtALY3* (At1g66260) *AtALY4* (At5g37720)	TREX complex adaptor protein	Dufu et al. (2010) Pendle et al. (2005) Uhrig et al. (2004) Yelina et al. (2010)

## TAF15b

RNA polymerase II pre-initiation complex assembly requires the presence of general transcription factors (GTFs). Transcription factors for polymerase IID (TFIID), one of these GTFs, comprises a complex of several subunits that include TATA-box binding protein (TBP) and TBP-associated factors (TAFs). The combination of these proteins makes up the GTF TFIID, the major core promoter recognition factor that provides scaffolding for the assembly of other GTFs. TAFs can bind activators and other transcriptional regulators; some of them exert catalytic activity modifying the histone code and transcriptional regulators. For example, TAF1 alone displays kinase, acetyltransferase, and ubiquitin-activating/conjugating enzyme activity, all in one protein. TAF1 can de-condense chromatin by H3–H4 histone acetylation and ubiquitination of histone H1; with its two kinase domains, it also directly phosphorylates some transcription factors (Dikstein et al., 1996; Mizzen et al., 1996; Pham and Sauer, 2000). Mining the *Arabidopsis* genome identified 18 putative TAFs, including TAF15a and TAF15b (Lago et al., 2004). AtTAF15b (**Table 1**) is the *Arabidopsis* homolog closest to the TAF protein that interacts in human cells with CIP29. Although some TAFs, e.g., TAF1, have been characterized in *Arabidopsis* (Bertrand et al., 2005; Benhamed et al., 2006), TAF15b has not. Since TAF15b does not contain any conserved catalytic domain that would provide insight into its putative molecular function, we investigated whether it has an effect on mRNA export. Whole mount *in situ* mRNA localization on two T-DNA lines (Salk_061974 and Sail_35_B06), inserted 17 bp apart in the third intron of At5g58470, did not reveal any significant changes in the amount of nuclear mRNA compared to wild type plants (unpublished). Thus, it is likely that TAF15b is not absolutely required for mRNA export under our assay conditions or that it is needed only for export of certain mRNAs, which is not possible to detect with poly-A mRNA localization. We are currently investigating the putative role of TAF15b in innate plant immunity and other cellular functions.

## DEAD-BOX RNA HELICASE

Employing BlastP with CIP29-interacting RNA helicase, we identified two *Arabidopsis* proteins (At5g11170 and At5g11200) with identical e-values. Both helicases are separated by only two genes on chromosome 5 and show 100% identity at the amino acid level; neither has been studied in plants. Previous mining of *Arabidopsis* sequences unveiled the presence of 32 DEAD-box RNA helicases in *Arabidopsis* (Aubourg et al., 1999). Based on the nomenclature proposed by them, At5g11170 is AtRH15 (**Table 1**). However, At5g11200 was not identified by Aubourg et al. (1999), perhaps due to its high degree of similarity to At5g11170. Therefore, we suggest re-naming At5g11200 as AtRH15b. The new TAIR (The Arabidopsis Information Resource) annotation identifies At5g11170 and At5g11200 as homologs of human UAP56. Alternate UAP56 names are DDX39 and BAT1, and we refer to UAP56 as DDX39 since it has been linked to CIP29 as the human DDX39. Enzymes that tap into energy released by the hydrolysis of a nucleotide triphosphate to unwind double-stranded RNAs are defined as RNA helicases (de la Cruz et al., 1999), linking them to every possible step in mRNA biogenesis and function. In the context of mRNA export, the unwinding activity of helicases can make mRNA more or less accessible to mRNA-processing proteins, thereby facilitating the addition and removal of proteins, and are involved in mRNP remodeling at the NP basket site. As mentioned previously, CIP29 is known to amplify the RNA-unwinding activity of DDX39 (Sugiura et al., 2007). The precise roles of AtRH15 and AtRH15b are unknown, and since their amino acid sequences are identical, it is likely that they complement each other functionally. It would be interesting to assess whether MOS11 can increase the RNA-unwinding activity of AtRH15 and/or AtRH15b as CIP29 does for DDX39. It should be mentioned that AtRH15 (like many helicases) can be sumoylated (Meier, 2012).

Very few functional analyses of DEAD-box RNA helicases have been conducted in *Arabidopsis*. Genetic screening of plants showing cold sensitivity identified *LOS4* (LOW EXPRESSION OF OSMOTICALLY RESPONSIVE GENES4; Gong et al., 2002), which encodes a DEAD-box RNA helicase that localizes to the nucleus and cytoplasm. *los4-1* mutant plants are very sensitive to chilling temperatures, particularly in the dark. LOS4 has also been shown to be required for mRNA export in a temperature-dependent manner, and LOS4–GFP (green fluorescent protein) fusion accumulates at the NE (Gong et al., 2002, 2005) where it may be involved in mRNP remodeling.

In human cells, the helicase DEAD-box protein 5 (Dbp5) is known to be required for the directionality of mRNA export (Tran et al., 2007). Dbp5 accumulates on the cytoplasmic side of the NE where it removes Nab2 from mRNP (Tran et al., 2007). Export functions as a cargo:carrier complexe; once mRNA has reached the cytosol, the carrier is disassembled to prevent cargo re-entry from the destination compartment (Stewart, 2010). The helicase that controls directionality in plants is unknown. However, LOS4 is the *Arabidopsis* helicase with the strongest sequence similarity to Dbp5; it accumulates at the nucleus outer membrane and is observed in the cytoplasm and nucleus (Gong et al., 2005). Two other helicases – *STRS1* and *STRS2* (*STRESS RESPONSE SUPPRESSOR*) corresponding to AtRH5 (At1g31970) and AtRH25 (At5g08620), respectively – have been shown to induce tolerance to salt, osmotic and heat stresses, suggesting that helicases suppress responses to abiotic stress (Kant et al., 2007). However, it is not known whether these helicases are involved in mRNA export. More than 30 helicases have been identified in *Arabidopsis* and it is conceivable that one helicase or a specific group of helicases may have specificity for groups of transcripts such as transcripts induced after biotic stress.

## ALY

Counterparts of the metazoan *ALY* (also known as *REF*, *BEF*, and *THOC4*) and its *Saccharomyces cerevisiae* homolog *YRA1* exist in plants (**Table 1**); as in animals, the number of ALY-coding genes in plants varies by species (Uhrig et al., 2004; Dufu et al., 2010). The exact functions of plant ALYs are not yet known. Four *ALY* genes have been reported so far in *Arabidopsis thaliana*: At5g59950, At5g02530, At1g66260, and At5g37720 (hereafter referred to as *AtALY1*, *AtALY2*, *AtALY3*, and *AtALY4*; Uhrig et al., 2004; Pendle et al., 2005; Yelina et al., 2010). Despite the relatively moderate sequence similarity between AtALYs (55–71%), AtALY1, AtALY2, AtALY3, and AtALY4 respectively share 41, 42, 38, and 48% sequence identity with human ALY. All four AtALYs localize to the nucleoplasm and, with the exception of AtALY2, also accumulate in the nucleolus, a poorly studied compartment in plants (Uhrig et al., 2004; Pendle et al., 2005; Shaw and Brown, 2012). Microarray data from Genevestigator (https://www.genevestigator.com/gv/) indicate medium to high expression levels for the four *AtALY* genes in seedlings, inflorescences, and shoots; lack of expression data for other tissues, however, makes it difficult to determine whether or not *AtALY* gene expression is tissue-specific.

Current models of mRNA export in animals and yeast depict ALY as a conserved component of TREX complex along with THO subcomplex, UAP56 (Sub2 in yeast), TEX1, and CIP29 (Tho1 in yeast), as a *bona fide* component (Carmody and Wente, 2009; Dufu et al., 2010; Chi et al., 2012; Katahira, 2012). However, recruitment of TREX complex components to mRNAs appears to proceed via distinct mechanisms in metazoans and yeast, possibly because of differences in gene structure, as the yeast genome contains far less introns than animal genomes (Cheng et al., 2006). Briefly, ALY and UAP56 are recruited to mRNA in an interdependent manner; UAP56 is then displaced by the Nxf1–Nxt1/TAP–p15 (Mex67–Mtr2 in yeast) heterodimer which is involved in mRNP targeting of nuclear pore complex (NPC; Carmody and Wente, 2009; Dufu et al., 2010; Walsh et al., 2010; Chi et al., 2012; Katahira, 2012). In human cells, TREX complex is recruited mostly in a splicing- and cap-dependent manner and binds near the 5′ end of spliced mRNA through interaction between ALY and the cap-binding complex protein CBP80. This mRNA 5′-end positioning of TREX complex is thought to account for the 5′–3′ orientation of the mRNA molecule during its export (Cheng et al., 2006; Kohler and Hurt, 2007).

Experimental results on *Arabidopsis* support the hypothesis that THO/TREX complex is conserved not only in yeast and metazoans, but also in plants, and strongly indicate a role for this complex (or at least some of its components) in plant immunity (Furumizu et al., 2010; Yelina et al., 2010; Pan et al., 2012b). For example, AtTEX1 and AtTHO1 have been shown to be required for the biogenesis of a subset of *trans*-acting small interfering RNAs (tasiRNAs; Yelina et al., 2010). Interestingly, it is now known that, in Fabaceae and Solanaceae, tasiRNAs mediate nucleotide-binding leucine-rich repeat gene silencing in the absence of pathogen threats (Zhai et al., 2011; Li et al., 2012). Furthermore, AtHPR1, a component of THO subcomplex, was recently identified as a mediator of disease resistance. *hpr1-4* mutant plants display compromised mRNA export and lack basal resistance to virulent bacterial and fungal strains (Pan et al., 2012a). To our knowledge, HPR1 is the only free nuclear protein (not bound to the NE) known to affect both mRNA export and basal defense responses.

*Arabidopsis* and *Nicotiana benthamiana* ALY proteins have been found to interact with P19 silencing suppressor protein of *Tomato bushy stunt virus* (Uhrig et al., 2004; Canto et al., 2006). ALY–P19 interaction results in the relocalization of AtALY2, AtALY4, and two *N. benthamiana* ALY proteins from the nucleus to the cytoplasm; however, it also leads to accumulation of P19 in the nucleolus by those ALY proteins that do not re-localize to the cytoplasm (Uhrig et al., 2004; Canto et al., 2006). Interestingly, nucleolar targeting of P19 interferes with its silencing suppression activity (Canto et al., 2006). Unfortunately, the biological role of ALY–P19 interaction is still unknown.

Yeast two-hybrid and *in vitro* findings have raised the assumption that AtALY3 and AtALY4 likely interact with the nuclear enzyme poly(ADP-ribose) polymerase 1 (PARP1; Storozhenko et al., 2001). Although this interaction has not been demonstrated *in planta*, it is noteworthy that plant PARPs have been reported to mediate some of the immune responses triggered upon recognition of microbe-associated molecular patterns (Adams-Phillips et al., 2010).

In mammals, ALY is emerging as a versatile protein involved in processes other than mRNA export. Thus, it is thought to stabilize some viral transcripts independently of their export (Stubbs et al., 2012). Furthermore, misregulation of ALY expression has been associated with tumorigenesis (Dominguez-Sanchez et al., 2011). In plants, such versatility would be consistent with the multiplicity of *ALY* genes and the heterogeneity of ALY protein subnuclear distribution.

## CONCLUDING REMARKS

This review highlights several similarities between the mechanistic aspects of animal and plant mRNA export processes. However, key questions remain to be answered that could promote understanding of plant mRNA export specificities. Which helicase is driving the directionality of export in plants? Does MOS11 possess helicase activity-enhancing capacity? What is the true role of the different ALYs, why are there four ALYs in plants, what role do they play in the nucleolus, can ALY bind suppressors of silencing other than P19? Plant nuclear proteome dynamics is still largely unknown, even more so the nuclear proteome of biotic or abiotic stressed plants. Although confocal imagery and genetics will remain core tools in resolving these issues, thorough proteomics analysis could lead geneticists on the right track. The fact that several pathogen virulence factors appear to be targeting the nucleus, combined with the observed high level of conservation of the mRNA export machinery suggest that the export machinery would represent a good target for pathogen effectors.

## Conflict of Interest Statement

The authors declare that the research was conducted in the absence of any commercial or financial relationships that could be construed as a potential conflict of interest.
